# Challenges and Facilitators in Implementing Remote Patient Monitoring Programs in Primary Care

**DOI:** 10.1007/s11606-023-08557-x

**Published:** 2024-04-23

**Authors:** Ruth Hailu, Jessica Sousa, Mitchell Tang, Ateev Mehrotra, Lori Uscher-Pines

**Affiliations:** 1 Department of Health Care Policy, Harvard Medical School, Boston, MA USA; 2 RAND Corporation, Arlington, VA USA; 3 Beth Israel Deaconess Medical Center, Boston, MA USA

## Abstract

**Background:**

The COVID-19 pandemic resulted in greater use of remote patient monitoring (RPM). However, the use of RPM has been modest compared to other forms of telehealth.

**Objective:**

To identify and describe barriers to the implementation of RPM among primary care physicians (PCPs) that may be constraining its growth.

**Design:**

We conducted 20 semi-structured interviews with PCPs across the USA who adopted RPM. Interview questions focused on implementation facilitators and barriers and RPM’s impact on quality. We conducted thematic analysis of semi-structured interviews using both inductive and deductive approaches. The analysis was informed by the NASSS (non-adoption and abandonment and challenges to scale-up, spread, and sustainability) framework.

**Participants:**

PCPs who practiced at least 10 h per week in an outpatient setting, served adults, and monitored blood pressure and/or blood glucose levels with automatic transmission of data with at least 3 patients.

**Key Results:**

While PCPs generally agreed that RPM improved quality of care for their patients, many identified barriers to adoption and maintenance of RPM programs. Challenges included difficulties handling the influx of data and establishing a manageable workflow, along with digital and health literacy barriers. In addition to these barriers, many PCPs did not believe RPM was profitable.

**Conclusions:**

To encourage ongoing growth of RPM, it will be necessary to address implementation barriers through changes in payment policy, training and education in digital and health literacy, improvements in staff roles and workflows, and new strategies to ensure equitable access.

## INTRODUCTION

The COVID-19 pandemic drove widespread adoption of many forms of telehealth across the USA, including remote patient monitoring (RPM). RPM focuses on the automated transmission of patient physiological measurements (e.g., blood pressure, blood glucose levels) to clinicians.^1,2^ The expectation is that the patient’s clinician monitors the patient’s data on a regular basis, possibly daily, and uses that data to adjust medications and detect complications. RPM can increase access and improve engagement in care by supporting frequent interactions with clinicians and longitudinal care outside of traditional office visits, thereby facilitating better chronic illness management. Studies have shown that RPM improves management of chronic conditions,^3^ such as hypertension,^4^ and reduces hospital readmissions among patients hospitalized for COVID-19.^5^

Medicare and most private insurers reimburse clinicians for RPM, and its usage accelerated during the pandemic.^6^ For RPM be eligible for reimbursement, patients use a device (e.g., blood pressure cuff, glucometer) that automatically transmits data. As of March 2021, RPM claims had increased four-fold compared to their pre-pandemic levels.^7^ RPM is reimbursed via CPT codes 99453 for onboarding, 99454 for at least 16 days of measurement transmission per month, and 99457/99458 for 20-min increments of monthly monitoring and interactive communications with the patient about RPM data. To date, the vast majority of RPM use has been utilized by primary care physicians (PCPs) for the management of hypertension.^7^

Although RPM’s growth has been substantial, analyses of claims data suggest that utilization has been concentrated among a minority of PCPs.^8^ In a 2022 American Medical Association Survey, 80% of physicians used synchronous video visits while only 30% used RPM.^9^ It is unclear why more PCPs have not adopted RPM, particularly in comparison to other forms of telehealth. Existing studies have explored RPM implementation challenges in a single program or health system.^10–14^ To address this gap in the literature, we conducted semi-structured interviews with 20 PCPs across the USA to describe the barriers to RPM adoption within primary care.

## METHODS

### Study Participants and Sampling Strategy

From September 19, 2022, to December 16, 2022, we conducted interviews with 20 PCPs who had implemented RPM in their practices. We conducted criterion sampling, selecting participants based on a predetermined set of criteria. To be eligible, participants had to be PCPs who practiced at least 10 h per week in an outpatient setting, served adults, and were using automatic RPM (the model eligible for reimbursement by Medicare) to monitor blood pressure and/or blood glucose levels with at least 3 patients. We defined automatic RPM as the regular, automatic transfer of physiologic data to a patient’s PCP. In this model, patients are given a device that they use at home that automatically transmits data through a Bluetooth or cellular connection. PCPs who utilized other types of RPM (e.g., models requiring patients to manually enter data into a patient portal) were excluded. We also excluded physicians who worked in the Military Health System, Veterans Health Administration, Indian Health Service, and/or Kaiser Permanente Group.

To recruit, we advertised the study opportunity to an online research panel with over 785 thousand US physicians. This panel has been used in many federally funded research studies and is comprised of physicians who have joined an online platform to access clinical content (news, condition and drug information, and journal articles), continuing medical education activities, and clinical tools.^15,16^ Upon joining the platform, physicians are given the option to opt-in to be contacted regarding research opportunities. Approximately 2000 PCPs in the panel were emailed information about the study opportunity and invited to complete a nine-item screener to assess eligibility for participation. Drawing from the respondents who met inclusion criteria, we invited 32 PCPs to participate in a 60-min interview using purposive, heterogeneity sampling. Our goal was to have a final sample that varied on key characteristics such as region, type of RPM, and physician demographics. Recruitment continued until we reached thematic saturation, defined as the point at which additional interviews did not uncover new themes or patterns.

Interviews followed a semi-structured protocol that was informed by prior research on the implementation of digital technologies in healthcare settings. It included questions on the following topics: (1) details on practice setting and patient population; (2) basic details of their RPM program (i.e., number of participants, motivation); (3) details on their RPM workflow (i.e., patient selection, onboarding, data receipt, and review); (4) barriers encountered in establishing and maintaining RPM; (5) perceived impact of RPM on quality of care; (6) feedback from patients and staff; (7) details on reimbursement and financial impact; and (8) future plans for RPM. Three members of the study team (RH, LUP, JS) trained in qualitative research conducted the interviews. Interviews were recorded and transcribed. Participants were given a $175 gift card for their participation, and they provided verbal informed consent. This study was approved by RAND’s Institutional Review Board.

### Analysis

We conducted a thematic analysis using both inductive and deductive approaches informed by the NASSS (reasons for non-adoption and abandonment and challenges to scale-up, spread, and sustainability) framework.^17,18^ This framework is a synthesis of theories on technology implementation and has been used in multiple studies evaluating health systems’ implementation of digital health technologies.^18,19^ According to the NASSS framework, the degree of complexity across seven key domains may predict the likelihood of successful implementation of a digital health intervention: (1) clinical and sociocultural aspects of the targeted health condition, (2) features and nature of the technology, (3) value proposition, (4) characteristics of the adopter system, including staff and patients, (5) organizational capacity/changes to team interactions needed for adoption, (6) the wider political and regulatory system, and (7) interactions between domains and adaptation of the intervention over time.^18^

The lead author (RH) developed the initial codebook using codes that aligned with the NASSS framework and interview guide, and codes were discussed, refined, and finalized in group meetings among three members of the study team (RH, JS, LUP). The lead author then coded all transcripts using Dedoose data analysis software.^20^

The NASSS framework guided the analysis and presentation of results. While our study team considered all domains in the NASSS framework, only a subset of domains emerged as themes identified by PCPs in our sample.

## RESULTS

### Participant Characteristics

Twenty PCPs representing 13 different states participated in interviews. Participants worked in solo practices (40%), group practices (30%), hospital-based outpatient clinics (5%), FQHCs (15%), and other outpatient settings, including university student health centers (10%). Most participants (65%) started their RPM programs after 2020. The majority of participants (60%) reported having more than 20 patients in their RPM program (Table 1).

**Table 1:** Participant Characteristics

Characteristic	*N* (%)
Region	
Northeast	9 (45%)
South	5 (25%)
Midwest	2 (10%)
West/Pacific	4 (20%)
Primary practice setting	
Solo private practice	8 (40%)
Non-hospital-based group private practice	6 (30%)
Hospital-based outpatient clinic	1 (5%)
Community health center (e.g., FQHC)	3 (15%)
Other outpatient setting	2 (10%)
Years of RPM implementation	
< 3 years (post-COVID-19)	13 (65%)
> 3 years (pre-COVID-19)	7 (35%)
Number of patients in panel on RPM*	
≤ 20	8 (40%)
21–100	5 (25%)
> 100	7 (35%)
Type of RPM**	
Blood pressure monitoring	17 (85%)
Continuous glucose monitoring	16 (80%)
Other (e.g., weight)	3 (15%)

*For six PCPs who reported the percentage of patients on RPM, we used a panel size of 2000 to estimate the number of patients in the panel on RPM

**This column does not add up to 100% because some physicians were doing more than one type of RPM

The RPM programs varied on many characteristics related to NASSS domains, including the target condition (i.e., program inclusion and stopping criteria), aspects of the technology (e.g., use of alerts, integration with EHR), factors influencing value proposition (e.g., billing practices), and nature of the adopter system (e.g., role changes required of PCPs or staff). Table 2 summarizes some of the areas of variation, organized by NASSS domain. Themes presented below highlight some of the most salient barriers, and their corresponding NASSS domains, that may be inhibiting greater uptake of RPM.

**Table 2:** Variation in RPM Programs by NASSS Domain

RPM program domains	Examples of variation
Domain 1: The condition or illness	
Program inclusion criteria	Participants needed to decide how large they wanted their programs to be (i.e., how many patients they could monitor at one time) and the criteria for participation. Some PCPs invited all patients with hypertension or type 1 diabetes to participate. Others targeted patients with uncontrolled hypertension/diabetes or patients who had recently been hospitalized.
Duration of participation	Participants needed to determine how long patients would be monitored and criteria for termination from the program. Some kept patients on RPM indefinitely. Others limited the time on the program (e.g., for 6 months or until the patient’s blood pressure was controlled).
Domain 2: The technology	
Receipt of data	Participants needed to select the platform through which they would receive physiologic data. Most began using an independent platform or app where clinic staff could view the data and receive alerts. Others, typically those who had been using RPM for longer, were able to integrate RPM data into their electronic health record (EHR). While this initially required a significant time investment, integration into the EHR ultimately made RPM easier to manage.
Domain 3: The value proposition	
Billing	While some participants billed Medicare and other payers for monthly RPM monitoring, others did not. Some participants felt that RPM programs could generate revenue through the scheduling of additional telemedicine and in-person visits (i.e., to discuss RPM data) rather than, or in addition to, monthly monitoring. Others implemented RPM to improve quality and were not particularly focused on increasing revenue.
Domain 4: The adopter system (changes in staff roles and practices; expectations for patients)	
Management of patient communication	RPM programs must onboard patients (i.e., set them up with a device and teach them how to use it), provide on-demand technical support, ensure compliance with monitoring, and review incoming data. Some participants outsourced these tasks to a vendor or other third party (e.g., different departments within health system), while the majority had their own clinic staff manage all aspects of the program. Some participants managed the majority of tasks internally but used a vendor in a more limited capacity (e.g., only to provide technical support).
Review of data	Participants had to develop processes for reviewing data. In some cases, the PCP would be responsible for reviewing all incoming data on a daily or weekly basis. In other cases, PCPs developed a triage system where clinic staff or a vendor would review incoming data first and pass off time-sensitive alerts to the PCP to review (see “Organizational capacity” below). PCPs varied in how often they reviewed data. Some set aside time at the end of the day or on weekends to periodically review data. Others reviewed RPM data continuously as it came in. Others only reviewed data during their regularly scheduled visits with the patient. Some arranged regular telemedicine visits for the purpose of reviewing data.
Workflow for responding to alerts/abnormal readings	Participants had different approaches to responding to alerts (i.e., abnormal reading) and communicating with the patient. Some would initiate a telephone call with the patient or message them within the portal. Others who worked in group practices would ask their staff to set up a telemedicine or in-person appointment with the patient or refer them to the emergency department.
Requirements of patients	Some participants felt that RPM required strong digital and health literacy, while others felt that the requirements of patients generally seemed achievable and acceptable to all patients. Few participants discussed strategies or resources (e.g., digital navigators, care coordinators) for supporting patients with lower health or digital literacy.
Domain 5: The organization (available resources, changes to team interactions and routines)	
Organizational capacity	Participants discussed different strategies for managing the incoming data generated by RPM given competing demands in primary care. Some participants had the resources or staffing to establish new care pathways for managing RPM, such as by working with a medical assistance or nurse who served as the initial point of contact for incoming data and alerts.
Domain 6: The wider context	
Payment models	About half of participants billed payers for RPM services, while others who were salaried or working under capitation models had fewer incentives to bill insurance. Some only offered RPM to participants with Medicare or other types of insurance with predictable reimbursement.

### Theme 1: Lack of Digital and Health Literacy Among Patients

The fourth domain of the NASSS framework suggests that patients may be more likely to use a digital health technology if what is expected of them is both achievable and acceptable.^18^ According to participants, both digital and health literacy among patients were barriers to the use of RPM, limiting the number of patients who could successfully engage with RPM and making the onboarding process more time-consuming for PCPs.

Many participants felt that digital literacy was a prerequisite for patients’ participation in RPM. A physician in solo private practice stated, “There are probably people that I would deem not eligible because of their inability to participate with the technology, but they may otherwise benefit from the program.” PCPs felt that older patients in particular had difficulty with the more technical aspects of RPM (e.g., connecting a device to Bluetooth). Another physician in solo private practice targeted young adults for inclusion in RPM, explaining, “I think for me, it’s anywhere from 30- to 60-year-olds who are usually fairly well educated, tech savvy… and are willing to put in the time to learn how to deal with particular problems.”

However, some disagreed and felt digital literacy was not an issue because the devices were easy to use. Rather, they believed that the *perception* that RPM devices were difficult to use had inhibited some patients from trying RPM. “The equipment is very intuitive,” a provider from a solo private practice explained. “Some of the older patients who are not tech savvy, they have that reluctance because they feel like they won’t be able to use the equipment and it’s not going to serve a purpose.”

Many participants also considered a patient’s health literacy and whether they were familiar with and understood their disease. According to a physician who worked at a student health center within a university, “Regardless of what we’re doing and what [data] we’re obtaining, if they [patients] don’t understand the benefit of it and what it means and how it could play out productively in their life, the chances that anything’s really going to productively come from this is very slim.” Participants also observed that patients needed the time and motivation to engage with RPM. A physician from a community health center pointed out that he could not provide RPM (which required multiple visits and ongoing interactions with clinic staff) to patients less engaged with their care or who did not have the bandwidth to add RPM into their daily routines.

### Theme 2: Substantial Role Changes Required for Handling Incoming Data

The fourth domain of the NASSS framework suggests that there will be less adoption of digital health technologies which require staff to take on new tasks or change their scope of practice.^18^ Almost all participants agreed that it was challenging to implement RPM given competing demands faced by PCPs and their staff. From introducing patients to devices to finding time in the day to analyze incoming data, each step of the process could be burdensome. And while all participants ultimately found the additional information provided by RPM to be helpful in improving patient care, many found the volume of alerts, emails, and messages difficult to handle. As a physician from a university student health center explained, “If I’m being 100% honest… in the first couple of patients that I had in the first three or four weeks, it was the sit down come to God moment of, ‘Okay, I can’t keep getting these messages all day long, something’s got to give here.’”

Participants mentioned that they frequently had to work additional hours to review data, even in cases where supporting staff played a significant role in the process. Also, several participants explained that they reserved time at night or on weekends (vs. continuously) to review data. This strategy was necessary because participants were busy during work hours, and they also felt it was distracting to review RPM data throughout the day. As a physician from a group private practice explained, “[If you review RPM data] You can get diverted on a tangent and then lose your train of thought on the patient you just saw, or you might forget something. And that’s happened to me.”

### Theme 3: Organizational and Care Team Changes Required for Handling Incoming Data

The fifth domain of the NASSS framework suggests that there will be less adoption of digital health interventions which require significant organizational changes (e.g., to team routines or care pathways), particularly for organizations with fewer reserve resources.^19^ Participants described the workload associated with RPM as unpredictable, making the program difficult to integrate into a busy practice without surplus resources. The frequency and urgency of alerts could vary from day to day and from patient to patient. To help manage the incoming data and avoid unplanned interruptions to their schedules, some participants relied on clinic staff (e.g., medical assistants, nurses) to conduct an initial review of the data. Only urgent cases were then flagged for review by the physician. However, not all participants were able to involve other staff members in their workflows, either due to limited bandwidth or working in a solo practice.

To manage the data deluge, some participants only periodically reviewed RPM data to understand trends over time. Instead of reviewing data daily or weekly, they reviewed data only before a scheduled visit. According to a physician from a group private practice, “Most of the time, the readings are looked at every two to four weeks, unless there is a more urgent need to look at it on a day-to-day basis. But for most people, it’s to get a trend, and you’re going to want a longer timeframe, a duration to see where that trend is.”

Some participants also worked with vendors to reduce the burden on clinic staff. In such cases, vendors often handled the distribution of devices and onboarding of patients. In some cases, they also reviewed incoming data and escalated alerts to the practice. Some vendors even handled communications with the patient (e.g., to alert them to an abnormal reading or remind them to take their blood pressure to ensure compliance with Medicare’s requirements of a minimum of 16 readings per month). As a physician from a group private practice explained, “The benefit of having a vendor and having a monitoring team, is the patient has this 24/7 access to someone who’s monitoring their sugars. And I usually get involved where we start to notice very low levels, very high levels or we start to notice trends.”

### Theme 4: Weak Value Proposition for the Healthcare Organization

According to the third domain of the NASSS framework, there will be greater adoption of a digital health technology with a clear value proposition and evidence of clinical benefit.^18^ In general, participants agreed that RPM improved quality of care. Participants liked that RPM gave them a better understanding of their patients’ health and disease state. According to a PCP from a university student health center, “[With RPM] I know the movie of your health as opposed to pictures of your health.” The most common benefits of RPM mentioned by participants included that it led to more informed prescribing (e.g., the appropriate combination of medications, the right dose), and it reduced the amount of time required to get a patient’s hypertension or blood sugar under control. This is because PCPs were able to adjust medications based on nuances and fluctuations in a patient’s data they would not have noticed otherwise. The large majority of participants mentioned that they believe RPM has decreased hospitalizations and emergency department visits among RPM users in their practice. Some participants also mentioned that RPM led to greater patient motivation, engagement, and health literacy. For some patients, it also reduced the stress associated with in-person visits (since data was coming in regularly vs. only during office visits), provided “peace of mind” between visits, and increased the efficiency of visits. RPM also reduced fear of liability and increased participants’ confidence in their treatment plans.

However, these positive impacts on quality could not be realized without significant investments in time and resources. In addition, most participants did not think RPM was profitable. Although all the participants in the sample were eligible for reimbursement from Medicare, only about half billed any payers for RPM services. Some participants were not billing because they had no incentive to bill (e.g., were salaried, were under capitation). Others said that billing for RPM was not worth the effort (e.g., due to low reimbursement rate, low volume of patients). Several noted that the reimbursement rate was too low, given the amount of work RPM requires, or too limited, given that some participants monitored multiple vital signs but could only bill for the monitoring of one vital sign per patient.

Participants who found RPM to be lucrative often offered RPM to all their patients with diabetes or hypertension, rather than limiting services to patients with poor disease control. Some also only offered RPM to patients with Medicare insurance or other types of insurance with predictable reimbursement, feeling that this maximized sustainability. Several of these participants also planned to keep their patients on RPM indefinitely.

Some participants did not bill for RPM directly but found other ways to use RPM to increase revenue. Frequent (billable) telemedicine visits were used to check in with patients about the results of RPM and implications for their care. Participants believed these additional telemedicine visits replaced some in-person visits. Participants were split on whether RPM had a significant effect on the number of in-person visits they were delivering.

A few participants discussed that patient cost could be a barrier to broader utilization. For example, several participants had patients who dropped out of RPM because of high out-of-pocket costs. This may be less of an issue for more affluent patients. One physician at an FQHC noted that the patients who used RPM successfully were typically those with commercial insurance rather than Medicaid and had more health literacy.

## DISCUSSION

Our interviews with PCPs highlighted many possible reasons why uptake of RPM has been relatively modest. Although participants reported favorable views of RPM, in particular that it improved quality of care, PCPs emphasized challenges with patient digital and health literacy, the need for organizational restructuring to support RPM workflows, and the lack of a clear business case. Several of our findings echo prior studies on RPM implementation. Previous studies have also highlighted how workflow changes are needed to integrate RPM^10^ and the importance of patient health literacy.^21^

Our study suggests several paths forward to encourage greater adoption of RPM. First, perhaps PCPs should not always be the point person receiving RPM data. Participants often spoke about needing to manage incoming RPM data in the context of very busy primary care practices that are structured around patient visits. Taking time to deal with incoming alerts was burdensome and difficult to incorporate into existing workflows. Further, research has shown that management of this type of patient-generated data can contribute to physician burnout.^22^ To manage the data, some PCPs developed workarounds that Medicare likely did not intend in its billing guidance. For example, some physicians would schedule periodic visits to review data rather than review data as it came in. A more sustainable strategy may be to offload this task to vendors, clinic staff within their own practices, or specific RPM staff within a health system. Dedicated RPM staff could conduct around-the-clock monitoring of the data of patients across multiple practices and make routine medication changes, and only communicate with the patient’s PCP when necessary.

Second, our results highlight the need to develop inclusion criteria and patient supports for RPM to improve accessibility. Some participants did not offer RPM to older adults, to those without certain types of insurance, or to patients who they perceived did not have sufficient health or digital literacy. While these exclusions make sense from the perspective of the PCP for expediency, they may result in inequitable access to RPM. Given the higher risk among elderly patients and the potential for reduced contact with the healthcare system among those with lower medical literacy, PCPs are potentially missing populations who would most benefit from RPM. PCPs should consider offering RPM to every patient who meets predetermined clinical criteria and has educational resources and technical support (e.g., digital navigators) in place to address barriers to patient engagement. A number of toolkits and resources exist that provide practical guidance on how to implement digital navigation programs.^23^

Finally, our findings have implications for payment policy. At present, Medicare specifies that patients must transmit data for at least 16 days per month for a practice to receive reimbursement. However, while PCPs are compensated for time spent analyzing the data (in 20-min increments), policies do not specify how often PCPs must review incoming data or how they should respond to the data (i.e., simply observing the data vs. having to document making impactful treatment decisions based on the data). In prior research, characteristics of more effective RPM programs include targeting of high-risk populations and providing timely and responsive care through frequent monitoring.^24^ In our study, multiple clinicians offered RPM to all their patients and only checked the data at periodic visits. Payers could consider restricting use of RPM to patients with poor disease control or high risk for hospitalization and specifying the minimum frequency of clinician monitoring.

Our study had several limitations. First, interviews can be affected by social desirability bias and recall bias. Further, not all participants could provide details on the specific roles of staff or on program design (e.g., how often nurses were reviewing data, what platforms they were using). In addition, all participants in our sample were RPM adopters. We did not engage participants who considered implementing RPM and ultimately chose not to. Non-adopters may confront additional challenges. Furthermore, this study reflects barriers experienced by early adopters of RPM. Barriers may evolve as more clinicians and systems adopt RPM. Finally, participants could not speak to the patient experience.

Despite these limitations, our findings point to several significant barriers to high-value RPM adoption. It will be necessary to conduct research that determines the prevalence of these barriers. If similar barriers emerge as the most common, policymakers and payers should address them through changes in policy, training and education in digital and health literacy, improvements in staff roles and workflows, and new strategies to ensure equitable access.
